# Endothelial microparticles released by activated protein C protect beta cells through EPCR/PAR1 and annexin A1/FPR2 pathways in islets

**DOI:** 10.1111/jcmm.13191

**Published:** 2017-05-19

**Authors:** Guillaume Kreutter, Mohamad Kassem, Ali El Habhab, Philippe Baltzinger, Malak Abbas, Julie Boisrame‐Helms, Lamia Amoura, Jean Peluso, Blandine Yver, Zobairi Fatiha, Geneviève Ubeaud‐Sequier, Laurence Kessler, Florence Toti

**Affiliations:** ^1^ EA7293 Vascular and Tissular Stress in Transplantation Federation of Translational Medicine of Strasbourg Faculty of Medicine University of Strasbourg Illkirch France; ^2^ UMR7213 CNRS Laboratory of Biophotonics and Pharmacology Faculty of Pharmacy University of Strasbourg Illkirch France; ^3^ Department of Diabetology University Hospital CHU de Strasbourg 1 place de l'Hôpital Strasbourg Cedex France; ^4^ Department of Pharmacy‐sterilization University Hospital CHU de Strasbourg Strasbourg France; ^5^ UPS1401‐ Plateforme eBiocyte Faculty of Pharmacy University of Strasbourg Illkirch France; ^6^ Department of Anesthesia‐Reanimation University Hospital, CHU de Strasbourg, 1 place de l'Hôpital Strasbourg Cedex France

**Keywords:** Islets transplantation, microvesicles, activated protein C, annexin A1, endothelium, β‐cells, beta cells

## Abstract

Islet transplantation is associated with early ischaemia/reperfusion, localized coagulation and redox‐sensitive endothelial dysfunction. In animal models, islet cytoprotection by activated protein C (aPC) restores islet vascularization and protects graft function, suggesting that aPC triggers various lineages. aPC also prompts the release of endothelial MP that bear EPCR, its specific receptor. Microparticles (MP) are plasma membrane procoagulant vesicles, surrogate markers of stress and cellular effectors. We measured the cytoprotective effects of aPC on endothelial and insulin‐secreting Rin‐m5f β‐cells and its role in autocrine and paracrine MP‐mediated cell crosstalk under conditions of oxidative stress. MP from aPC‐treated primary endothelial (EC) or β‐cells were applied to H_2_O_2_‐treated Rin‐m5f. aPC activity was measured by enzymatic assay and ROS species by dihydroethidium. The capture of PKH26‐stained MP and the expression of EPCR were probed by fluorescence microscopy and apoptosis by flow cytometry. aPC treatment enhanced both annexin A1 (ANXA1) and PAR‐1 expression in EC and to a lesser extent in β‐cells. MP from aPC‐treated EC (eM_aPC_) exhibited high EPCR and annexin A1 content, protected β‐cells, restored insulin secretion and were captured by 80% of β cells in a phosphatidylserine and ANXA1‐dependent mechanism. eMP activated EPCR/PAR‐1 and ANXA1/FPR2‐dependent pathways and up‐regulated the expression of EPCR, and of FPR2/ALX, the ANXA1 receptor. Cytoprotection was confirmed in H_2_O_2_‐treated rat islets with increased viability (62% *versus* 48% H_2_O_2_), reduced apoptosis and preserved insulin secretion in response to glucose elevation (16 *versus* 5 ng/ml insulin per 10 islets). MP may prove a promising therapeutic tool in the protection of transplanted islets.

## Introduction

Protein C is the circulating zymogen of aPC, an anticoagulant serine protease generated at the endothelial surface by thrombin‐mediated cleavage of the bound PC 1. aPC exerts its dual property in the preservation of vascular integrity and function not only by limiting thrombin generation through the proteolytic inactivation of factors Va and VIIIa but also by acting as an endothelial cytoprotector triggering the protease‐activated receptor 1 (PAR‐1)‐mediated anti‐inflammatory and anti‐apoptotic pathways 2. When bound to its specific endothelial receptor (EPCR), aPC behaves as a PAR‐1‐biased ligand through a specific cleavage at arginine 46.

Because endothelial damage is a prime sensor of ischaemia–reperfusion and a potential inducer of procoagulant and pro‐inflammatory responses, several pre‐clinical and clinical studies have investigated the interest of aPC in organ failure and examined the mechanism by which aPC protects the vessel and therefore the organ perfusion and function 3. Notably, new engineered aPC molecules, lacking anticoagulant properties but with high cytoprotective abilities, have entered a clinical trial aiming at showing neuroprotective effects in ischaemic brain stroke 4, 5.

Pancreatic islet transplantation by portal vein infusion is a cell therapy often associated with an early and acute form of ischaemia–reperfusion termed IBMIR (Instant Blood‐Mediated Inflammatory Reaction) that causes the destruction of ~70% of the graft within the first 48 hrs 6. Ischaemia is the consequence of the islet isolation that abolishes blood supply, while islet infusion through the portal vein triggers reperfusion damages before islets’ full engraftment at the extremity of the secondary liver vessels 7, 8. Infused islets and residual exocrine cells from the islet preparation trigger platelet activation, thrombin generation, the initiation of the complement cascade, the production of ROS and cytokines by leukocytes recruited at the vicinity of the islets 7, 9, 10, 11. Under such pro‐inflammatory conditions, the expression of tissue factor (TF), the cellular initiator of coagulation, is induced in leukocytes, endothelial and also insulin‐secreting cells that all shed MP bearing the active form of TF thereby promoting coagulation close to the islets 10, 12, 13. MP are plasma membrane vesicles released in response to a variety of cellular stress like inflammation or apoptosis 14. MP expose or contain active proteins, lipids, mRNAs and act as cellular effectors between vascular cells or pancreatic cells 15, 16. Regardless of the eventual presence of TF, all MP are procoagulant because they expose phosphatidylserine, an anionic phospholipid that constitutes the catalytic surface for blood coagulation complexes and that potentiates TF activity 14.

The initial interactions of the islets with the hepatic endothelium and vascular cells are crucial to islet engraftment 17, 18. Indeed, the oxygen pressure decreases along the liver lobule making liver endothelial cells (EC) highly sensitive to oxidative stress and endothelial barrier exchanges pivotal for the maintenance of the microvessel function 19. After transplantation, the restoration of the endothelial lining of the intra‐islet capillary, which is mainly supported by the recipient's endothelial and progenitor cells, is key to islets’ revascularization and function. Indeed, the islet capillary network presents an important proportion of highly fenestrated microvascular ECs that are involved not only in blood supply to endocrine cells, but that also affect adult β‐cells function, that is insulin secretion 17, 18.

MP from progenitor ECs favour endothelial cell recruitment within the islet 20. The possibility of a MP‐driven cell crosstalk between endothelial and insulin‐secreting β‐cells within the liver vessels is supported by recent *in vitro* data showing that a suspension of MP and exosomes harvested from isolated islets modifies endothelial cell responses 17.

Most studies have examined the noxious MP properties and very few investigated their eventual beneficial effects. Interestingly, neutrophil and endothelial‐derived MP were identified as shuttles for annexin A1 (ANXA1), an anti‐inflammatory lipocortin possibly involved in MP‐driven cytoprotection 21, 22. ANXA1 is a 37‐kD member of the Ca^2+^ and phospholipid binding protein, superfamily that when secreted mainly binds to its formyl peptide receptors (FPR) 23, 24, 25. Interestingly, MP released from aPC‐treated ECs were reported cytoprotective. They bear the EPCR, the specific receptor of aPC, and deliver aPC to target ECs, thereby protecting them from pro‐apoptotic and inflammatory mediators released during septic shock 26, 27.

Previous studies have underlined the interest of aPC in the preservation of islets from ischaemia–reperfusion during transplantation 28, 29 and underline a possible contribution of MP to the aPC‐mediated beta cell cytoprotection. The mechanisms of aPC‐mediated beta cell protection within the islet, which can be considered as the smallest functional architecture of the pancreas, remain yet unknown.

The aims of this study were (*i*) to evaluate the protective effect of aPC on beta cells in a model of ischaemia/oxidative stress under conditions mimicking IBMIR, (*ii*) to evaluate the effects of endothelial‐ and β‐cell‐derived MP released upon aPC treatment on naive beta cell function and survival (*iii*) to identify the eventual underlying mechanism of aPC‐mediated beta cell cytoprotection.

## Materials and methods

### Rat β Cell culture

Rat β cells, Rin‐m5f (CRL‐11605™; ATCC, Manassas, VA, USA), were seeded at 125,000 cells/cm2 in RPMI 1640 (PAN™ Biotech GmbH, Aidenbach, Germany) medium containing 4.5 g/l glucose, 10 mM HEPES, 2 mM glutamine, 1 mM sodium pyruvate and supplemented with 10% foetal bovine serum (Gibco, Saint Aubin, France) and 20 μg/ml gentamycin (Lonza, Basel, Switzerland). Cells were cultured in 12‐well culture plates at 37°C and 5% CO_2_ in a humidified atmosphere.

### Islets isolation

Rat islets were isolated by 1 mg/ml collagenase treatment (type XI; Sigma‐Aldrich, L'isle d'Abeau Chesnes, France) followed by density gradient separation (Ficoll; Sigma‐Aldrich) as previously described 30.

Harvested islets were washed in RPMI‐1640 medium containing 2 g/l glucose (PAN™ Biotech GmbH) and hand‐picked. For optimal recovery of functional islets, each hundred recovered was maintained for 12 hrs in RPMI‐1640 supplemented with 10% foetal calf serum, penicillin/streptomycin (100 UI/ml), in a Petri dish placed under humid 5% CO_2_ atmosphere at 37°C before any experimental procedure.

### Primary coronary artery EC

Pig hearts were collected from the local slaughterhouse (COPVIAL, Holtzheim, France), and primary coronary ECs were isolated from the left circumflex coronary arteries as previously described 31. Briefly, left circumflex coronary arteries were excised from fresh heart, cleaned of adhesive conjunctive tissues, and the remaining blood was flushed with cold phosphate‐buffered saline (PBS) without calcium. ECs were isolated by collagenase treatment (type I, Worthington Biochemicals Corp., Lakewood, NJ, USA) at 1 mg/ml for 12 min. at 37°C. ECs were cultured in Petri dishes containing MCDB 131 medium (Life Technologies SAS, St Aubin, France) and 15% foetal calf serum supplemented with penicillin (100 U/ml), streptomycin (100 U/ml), amphotericin B (250 mg/ml) and L‐glutamine (2 mM, all from Lonza, Levallois‐Perret, France) and grown for 48–72 hrs (passage 0).

### MP generation, harvest and quantification

MP were harvested under sterile conditions from the supernatants of either young P1 ECs (eMP_aPC_) or RIN‐m5f β cells (βMP) submitted to a 70 nM aPC treatment during 24 hrs (Xigris^®^, Lilly). Floating apoptotic cells and debris were first discarded (800 g 15 min.) and MP washed in Hanks balanced salt solution (HBSS) and concentrated by a double‐centrifugation step (14,000 *g*, 1 hr). Washed MP were kept at 4°C for not more than 3 weeks. For control purposes, endothelial MP (eMP_CTRL_) were harvested from the supernatant of untreated ECs and isolated using the same procedure.

Total MP concentration was determined by prothrombinase assay as previously described. Briefly, MP captured onto insolubilized annexin A5 were incubated with blood clotting factors (FXa, FVa, FII) and CaCl2. Conversion of prothrombin to thrombin was revealed by a chromogenic substrate, using a spectrophotometric reader set at 405 nm. Results were expressed as nanomolar PhSer equivalent (nM PhSer eq.) by reference to a standard curve constructed using liposomes of known concentration and PhSer eq. proportion 14.

### Endothelial and β cell MP‐mediated crosstalk under oxidative stress in cells and islets

Rin‐m5f rat insulin‐secreting cells were chosen as an adequate model for the study of the β cell response to prolonged oxidative stress and hyperglycaemia. Indeed, although not responsive to a short metabolic raise by glucose stimulation, Rin‐m5f develop apoptosis after prolonged exposure to H_2_O_2_ 32.

When endothelial or Rin‐m5f cells reached 70% of confluency, they were treated by 100 μM H_2_O_2_ to mimic the conditions of oxidative stress during islet ischaemia (Sigma‐Aldrich), as reported elsewhere 13. Cell supernatants were collected 24 hrs after induction of the oxidative stress, MP isolated as above and the MP‐depleted supernatant was kept at 4°C for less than 1 month under sterile conditions for control purposes.

In MP‐mediated crosstalk cellular models, endothelial or β‐cell‐derived washed MP were pre‐incubated with Rin‐m5f during 6 hrs before H_2_O_2_ treatment (100 μM). In some experiments, pre‐treatment by 50 nM aPC was performed 4 hrs before application of the oxidative stress eventually combined to eMP.

MP‐mediated crosstalk within the islets was assessed 12 hrs after their isolation and culture in RPMI containing 2 g/l glucose and supplemented with 10% foetal calf serum. 20 nM PhtdSer eq. eMP or eMP_APC_ were added to each 20 islet suspension, 12 hrs before addition of 100 μM H_2_O_2,_.

In some crosstalk experiments, signalling pathways were inhibited by pharmacological treatment of the MP‐targeted cells. Phosphoinositide 3 kinase A (PI3K) was inhibited by 10 μM LY294002 (Tocris, Lille, France) incubated 1 hr prior addition of endothelial‐derived MP (20 nM PhSer eq.). Similarly, PAR‐1 was blocked by pre‐incubation with 10 μg/ml ATAP2 antibody (Santa Cruz, Dallas, Texas, USA) as previously described 33. Inhibition of the ALX/Formyl peptide receptor 2 (FPR2) was performed by continuous exposure to 10 μM WRW4 (Tocris).

### Quantification of apoptosis in cell lineages and islets

Rinm‐5F cells were washed and permeabilized by a 70% ethanol solution at 4°C for at least 24 hrs. After three washing steps, cells were resuspended in a solution containing I‐A RNase A (Sigma‐Aldrich) for 15 min. at 37°C. Saturating concentration of propidium iodide (Sigma‐Aldrich) was applied (0.1 mg/ml) and the degree of apoptosis evaluated by the quantification of hypodiploid DNA by flow cytometry (Guava; Merck‐Millipore, Molsheim, France). Necrosis, early apoptosis and late apoptosis were defined by annexin A5/PI^+^, annexin A5/PI^−^ and annexin A5/PI^+^, respectively. A total of 5000 cells were acquired for each individual sample.

In some experiments, apoptosis was measured in isolated islets using a modified procedure. Briefly, 20 islets were treated by 100 μM H_2_O_2_ for 4 hrs. Islets were harvested and centrifuged (800 *g*, 5 min.) and further submitted to trypsin (Lonza) during 10 min. at 37°C before inactivation by addition of FCS allowing the complete dissociation of the islets’ and recovery of the constitutive cells. Islet cells were further pelleted and resuspended in RPMI medium without foetal serum. Double staining by FITC‐annexin A5 and propidium iodide (PI) was performed in the dark at 25°C for 15 min.(BD Biosciences, Franklin Lakes, NJ, USA). Apoptosis was measured by flow cytometry (Guava, Merck‐Millipore) using parameters set at linear gain as above.

### Insulin measurement

Insulin released in the conditioned medium of Rin‐m5F cells or in islet suspension was assessed by ELISA according to the supplier recommendations using the Matrix protocol when foetal calf serum was present in the medium (ELISA Kit Rat/Mouse Insulin; Millipore).

### Kinetics of the endothelial MP capture by target beta cells and EPCR probing

Endothelial MP isolated from aPC‐treated ECs (eMP_APC_) were stained using the PKH26 red fluorescence lipid probe (Sigma‐Aldrich) as described elsewhere, 13 and washed. 20 nM PKH26‐stained MP were added to growing cells at 70 % confluency in fresh medium and incubated during 1–24 hrs. Cells were then washed, fixed in paraformaldehyde 4% and kept at 4°C before EPCR labelling or assessment of PKH26‐ stained target cells by flow cytometry (see below).

After three washings, a biotinylated anti‐EPCR antibody (SantaCruz; dilution: 1:100, 1 hrs, RT) was added to the suspension. Washed cells were incubated with FITC‐streptavidin (Sigma‐Aldrich; dilution: 1:150, 1 hr, RT) and DAPI solution (5 min., 300 nM) control conditions were untreated β‐cells (CTRL) and β‐cells incubated with the secondary antibody (data not show). After washing and strip mounting, cells were observed by fluorescent microscopy (Leica FW 4000, ×40 objective). At least nine random fields were analysed by sample. Results are expressed as the percentage of PKH26‐labelled β‐cells exhibiting the red MP fluorescence and as the green fluorescence intensity/β‐cells reflecting the degree of EPCR expression on β‐cells. Analysis was performed using ImageJ software (National Institute of Health, Bethesda, Maryland, USA).

Kinetics of the PKH26‐stained MP capture by target cells was also assessed by measurement of red fluorescence in cells using a flow cytometer set at logarithmic gain (Becton‐Dickinson, Pont‐de‐Claix, France). At least 5000 events were recorded for each sample.

### Pharmacological modulation of the interaction between MP and target cells

PKH26‐stained MP (20 nM) were incubated for 1 hr at 37°C with 10 μg/ml annexin A5, 20 μg/ml antibody against annexin A1, or vehicle and washed before their addition to target cells (70 % confluency) eventually pre‐incubated for 30 min. with 10 μg/ml WRW4, an inhibitor of FPR2. Red cell fluorescence was measured after 6 hrs. At least 5000 cells were acquired for each individual sample.

### Protein expression in target cells

After treatment, cells were washed twice with PBS and then lysed in TRIS buffer containing protease inhibitors (5 μg/ml leupeptin, 5 mM benzamidine) and 2% Triton^®^ X‐100 on ice. Total proteins (30 μg) were separated by electrophoresis on 10% SDS–polyacrylamide (Sigma‐Aldrich) gels as previously described (4). Blotting membranes (nitrocellulose, GE Healthcare, Amersham, UK, USA) were incubated with the different primary antibodies directed against mouse PAR‐1 (SantaCruz; 1:1000 dilution), rabbit cleaved caspase 3, mouse iNOS, mouse annexin A1 (Cell Signaling Technology, Danvers, MA, USA, 1:1000 dilution), FPR2/ALX (Abcam, cambridge USA, 1:1000) overnight at 4°C. Detection of β‐actin (Sigma‐Aldrich; 1:5000 dilution) or beta tubulin (Abcam; 1:1000) was used for normalization. After washing, membranes were incubated with the secondary anti‐mouse IgG antibody (Cell Signaling Technology, 1:10 000 dilution) at room temperature for 60 min. Pre‐stained markers (Invitrogen™, Carlsbad, CA, USA) were used for molecular mass determinations. Immunoreactive bands were detected by enhanced chemiluminescence (Amersham, GE Healthcare). Density analysis was performed using ImageQuant LAS 4000 imager (GE Healthcare).

### Measurement of aPC activity at cell and MP surface

The activity of aPC bound to the cell membrane or borne by the isolated MP and concentrated from the supernatant was assessed using a specific chromogenic substrate (S2366, Cryopep, Montpellier, France) as previously described 27. In brief, cells were washed three times and incubated with 0.75 mM S2366 and absorbance was measured at 37°C using a spectrophotometer equipped with a kinetics software (Versamax, molecular Device, UK). aPC activity was calculated by reference to a standard curve (Xigris^®^, 0.2–30 nM). Results are expressed as nM aPC/100,000 cells and nM aPC / 10 nM MP.

### Measurement of oxidative stress in beta cells

The intracellular ROS concentration was detected by the redox‐sensitive fluorescent probe dihydroethidium (DHE, Thermo Fischer, Illkirch, France), chosen for its high specificity for mitochondrial O_2_^−^. Cells were stained with DHE (5 μM) at 37°C in dark for 30 min. and examined by a fluorescence‐activated cell sorter (FACScan; BD Biosciences) with excitation length at 480–535 nm and emission at length 590–610 nm. ROS‐positive cells were detected by red fluorescence probing. At least 5000 cells were acquired for each individual sample 34.

### Islets viability

Twenty islets maintained in cell RPMI supplemented with 10 % SVF were treated during 6 hrs by 100 μM H_2_O_2,_ then washed by centrifugation (500 *g*, 5 min.) and incubated with fluorescein diacetate (0.67 μM) and propidium iodide (4 μM) before observation by fluorescent microscopy (Leica FW 4000, ×20 objective). Nine random fields were analysed per sample. Fluorescence intensity was analysed using the ImageJ software. Results are expressed as green intensity/islet surface unit. Data were obtained from four different islet preparations.

### Assessment of β‐cells function within the islets

Islets function was assessed by measuring insulin secreted in the suspension of 20 isolated islets challenged by a 10‐fold elevation in glucose concentration. Islets were pre‐incubated for 2 hrs in Krebs solution containing 100 nM HEPES, 0.5 mg/ml human albumin, 2.5 mM glucose. Aliquots from the incubation medium were taken to measure the insulin secreted at low glucose concentration. Islets were thereafter pelleted and re‐suspended in Krebs solution containing 25 mM glucose and medium aliquots were taken after 1 hr 30. All samples were stored at −20°C until insulin measurement by ELISA. Data are expressed as ng/ml of insulin per 10 islets.

### Statistical analysis

Data are expressed as mean ± standard error of mean (S.E.M.) and analysed using GraphPad Prism5^®^ Prism5 Graphpad Company, La Jolla, CA, USA. Statistical analysis between two groups was carried out using unpaired Student's *t*‐test. A *P* value <0.05 was considered significant. Experiments were performed at least in three separate experiments.

## Results

### aPC promotes the release of endothelial MP able to protect β‐cells against oxidative stress

**Figure 1:**
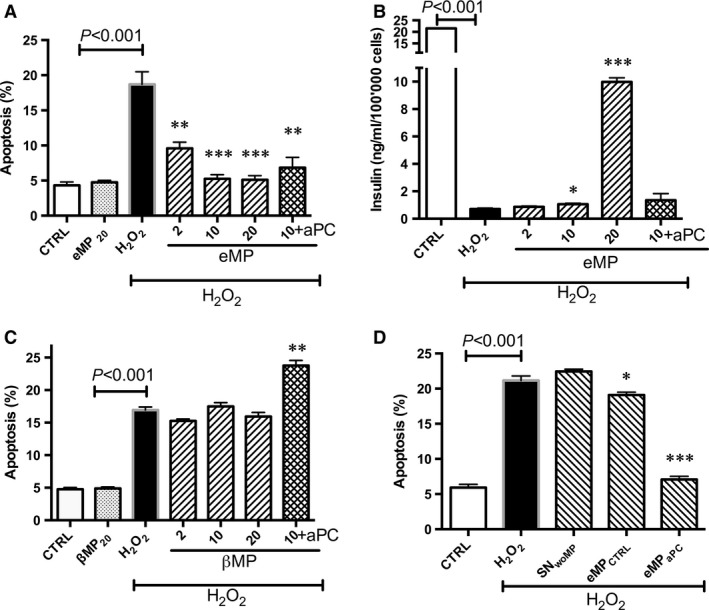
Effect of aPC alone or of aPC‐generated microparticles on β‐cells submitted to oxidative stress. (**A–B**) β‐cells were pre‐treated by aPC (70nM, 4 hrs) or endothelial cell‐derived MP treated by aPC (eMP_aPC_, 6 hrs) before the 24 h‐H_2_O_2_ treatment. Apoptosis was assessed by hypodiploid DNA labelling (**A**,* n* = 4). Insulin secreted in supernatant was measured by ELISA (**B**,* n* = 4). (**C**) β‐cells cells were pre‐treated with β‐cells‐derived MP (ßMP) during 6 hrs before treatment by oxidative stress, in the absence or presence of 50 nM aPC (aPC *n* = 4). (**D**) β‐cells were treated by the supernatant of control untreated endothelial cells (SN_woMP_) or by MP harvested from untreated resting endothelial cells (MP_CTRL_) during 6 hrs prior addition of H_2_O_2.._ Data expressed as mean ± S.E.M. (aPC, activated protein C; CTRL, untreated cells; eMP, microparticles isolated from aPC‐treated endothelial cells; βMP, microparticles from β‐cells treated by aPC; PhSer eq., Phosphatidylserine equivalent. *P < 0.05 *versus* H_2_O_2_; **P < 0.01 *versus* H_2_O_2_; ***P < 0.001 *versus* H_2_O_2_).

An incubation with 20 nM endothelial MP (eMP_aPC_) harvested from aPC‐treated ECs (ECs) prevented the H_2_O_2_‐induced β‐cells apoptosis. The degree of apoptosis was reduced by threefolds (18.7 ± 3.6% *versus* 5.1 ± 1.2 %, Fig. 1A). The eMP_aPC_‐mediated cytoprotective effect was confirmed by the prevention of the H_2_O_2_‐induced drastic drop in insulin secretion, concentrations in supernatant returning to significantly higher values from 0.7 ± 0.1 ng/ml/100,000 cells in H_2_O_2_‐treated β‐cells to 10 ± 0.5 ng/ml/100,000 cells (*P* < 0.001, *n* = 4, Fig. 1B). Of note, 20 nM eMP_aPC_ were sufficient to mediate a cytoprotective effect that was not observed in β‐cells treated by aPC alone (Fig. 1C)._._ Furthermore, 50 nM aPC had no additive effect to eMP_aPC_, suggesting a specific eMP_aPC_‐mediated cytoprotection_._ Importantly, aPC was not toxic to the β‐cells (unchanged viability and absence of apoptosis, data not shown).

Because the effector abilities of MP vary with the cell and the agonist that have initiated their generation, we examined whether beta cell‐derived MP (βMP_aPC_) generated by aPC treatment could also behave as autocrine effectors of β‐cells. Conversely to eMP_aPC,_ βMP_aPC_ had no protective effect (Fig. 1C). Interestingly, the comparison between endothelial MP harvested from naive ECs (eMP_CTRL_) and those generated from APC‐treated ECs showed that eMP_aPC_ were potent inhibitors of apoptosis, whereas prevention by eMP_CTRL_ remained negligible (18.2 ± 1% apoptosis in eMP_CTRL_‐treated *versus* 21.2 ± 0.7% in untreated H_2_O_2_‐stimulated cells, Fig. 1D). Interestingly, treatment of β‐cells by eMP‐depleted supernatants obtained after extensive centrifugation (14,000 g, 1 hr) did not lead to cytoprotection (MP+H_2_O_2_ :21. 2 ± 1.6% *versus* SN+H_2_O_2_ :19.1 ± 1), thereby excluding the effect of a truly soluble effector, such as cytokines or exosomes, eventually present in the conditioned medium, and confirming a specific MP‐driven response.

Altogether, these data indicate that endothelial‐derived MP are specific effectors of β‐cell cytoprotection and that treatment by aPC greatly enhances their ability.

### Kinetics of eMP_aPC_ integration by β‐cells and transfer of functional EPCR

**Figure 2:**
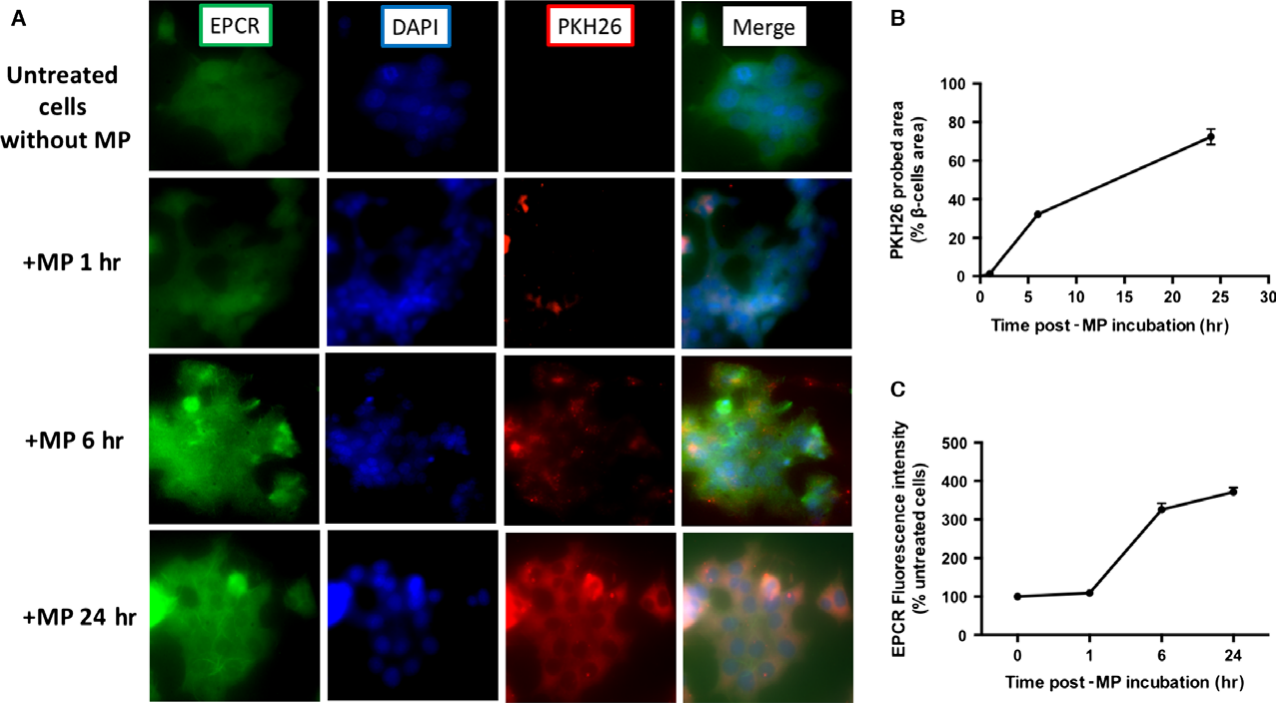
Integration of eMP_aPC_ by β‐cells and EPCR expression. β‐cells were incubated during 1, 6, 24 hrs with PKH26‐labelled endothelial eMP_aPC_ generated by aPC treatment, fixed and labelled with EPCR antibody and nuclei were labelled with DAPI. Representatives images of at least four independent experiments ×40 objective (**A**). (**B**) Integration of microparticles in β‐cells. Results are expressed as the percentage of PKH^+^ cells (*n* = 4). (**C**) EPCR expression by fluorescence intensity using FITC labelling EPCR antibody in β‐cells. Results expressed as fluorescence intensity/β‐cells normalized with control of untreated cells without MP. Data expressed as mean ± S.E.M. (*n* = 4). (aPC, Activated protein C; EPCR, Endothelial protein C Receptor; MP, Microparticles; PFA, paraformaldehyde).

PKH26‐labelled eMP_aPC_ were incubated with β‐cells during 1, 6 or 24 hrs. Their integration by the cell was probed by red fluorescence, and EPCR expression at β‐cells surface was simultaneously followed using an anti‐EPCR‐biotinylated antibody. After 6‐hrs incubation with eMP_aPC_, 32 % of the β‐cells layer area showed red fluorescence, reaching a significant 74% plateau after 24 hrs that represented the maximal capture of the MP by β‐cells (*n* = 4, *P* < 0.001 *versus* MP‐untreated cells, Fig. 2B). EPCR expression, revealed by green fluorescence at β‐cells surface, increased as early as 6 hrs after the addition of eMP_aPC_ (*n* = 4, *P* < 0.001 *versus* untreated cells, Fig. 2C). Green and red fluorescences followed a similar time‐curve, suggesting that eMP_aPC_ transfer EPCR to the target cell or early prompt its expression (Fig. 2).

**Figure 3:**
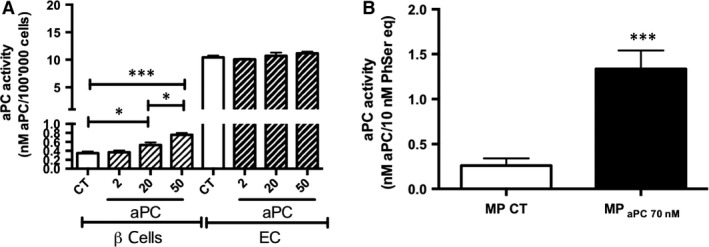
aPC activity at endothelial and β‐cell surface after 24 hrs treatment (**A**) and by aPC‐generated endothelial microparticles (**B**). Endothelial cells and β‐cells were treated by aPC during 24 hrs. Harvested endothelial MP were washed and concentrated and their quantity measured by prothrombinase assay. aPC activity was assessed using a S2366 chromogenic substrate. (**A**) aPC activity expressed as nM aPC/100,000 cells (*n* = 3‐4). (**B**) aPC activity expressed as nM aPC/10 nM PhSer eq. (*n* = 5). Data expressed as mean ± S.E.M. (aPC, activated protein C (Xigris^®^); CT, untreated cells; βMP, β‐cells; EC, endothelial cells; eMP, endothelial MP; MP, microparticles; PhSer eq., phosphatidylserine equivalent. *P < 0.05; ***P < 0.001).

At β‐cells surface, the presence of detectable amounts of a functional EPCR able to bind aPC was supported by the measurement of a low but concentration‐dependent aPC activity that remained, however, 10–15 times less than that quantified at the ECs surface (0.4 ± 0.1 eq nM aPC/100,000 β‐cells *versus* 10.5 ± 0.4 nM aPC/100,000 ECs, *P* < 0.001, *n* = 3, Fig. 3A). Similarly, a functional EPCR borne by eMP_aPC_ was evidenced. Compared to eMP_CTRL_ harvested from aPC‐untreated cells, MP_aPC_ bore a significantly ~ 20‐fold greater aPC activity (eMP_CTRL_ 0.2 ± 0.05 nM aPC *versus* eMP_aPC_ 1.46 ± 0.27 nM aPC, *P* < 0.001, *n* = 5, Fig. 3B).

### Interaction between eMP_aPC_ and β‐cells is a phosphatidylserine‐ and annexin A1‐dependent mechanism

**Figure 4:**
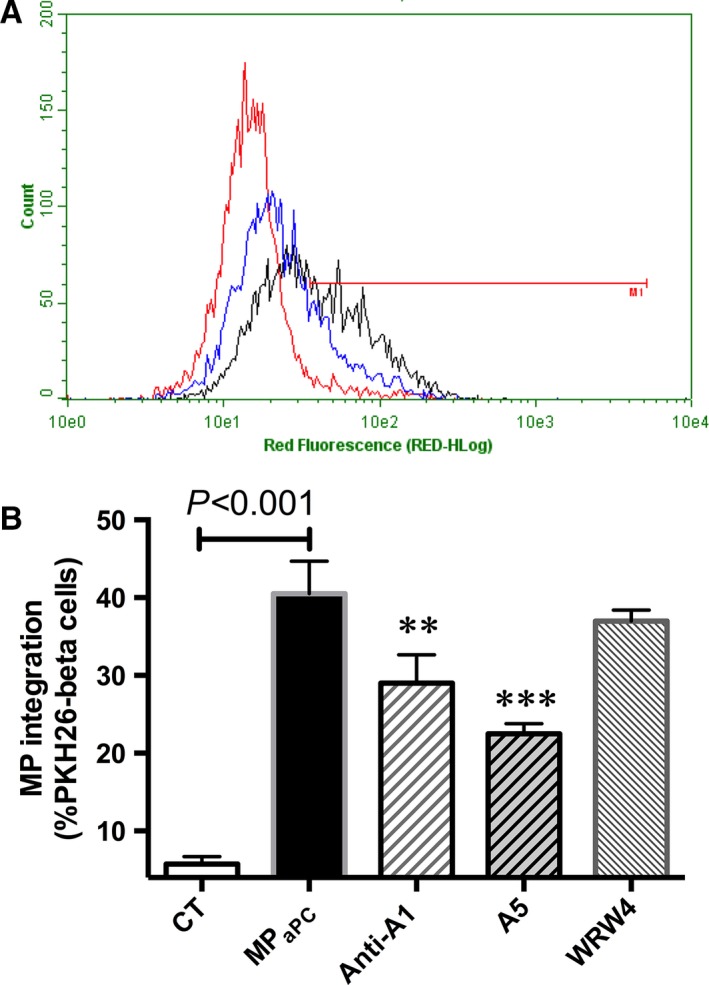
Mechanism of eMP_aPC_ integration by β‐cells. β‐cells pre‐incubated with or without WRW4 (10 μM 1 hr), a FPR2 antagonist were incubated during 6 hrs with PKH26‐labelled eMP_**aPC**_ pre‐treated or not by 10 μg/ml annexin A5 or 20 μg/ml annexin A1 antibody for 1 hr. Incorporation was assessed by flow cytometry. (**A**) Representative cytogram of beta cells without MP (red), with MP (black) or with annexin A5 (blue). (**B**) Data expressed as mean ± S.E.M., *n* = 4. (CT, untreated cells; eMP_aPC_, cells treated by endothelial microparticles released after aPC treatment. **P < 0.01 *versus* eMP_aPC_; ***P < 0.001 *versus* eMP_aPC_; †*P* < 0.001 *versus* H_2_O_2_+ eMP_aPC_).

To better understand the mechanisms supporting eMP_aPC_ and β‐cells interactions, PKH26‐labelled eMP_aPC_ were pre‐treated with annexin A5, a protein with high affinity for phosphatidylserine, WRW4, an antagonist to FPR2/ALX or with an antibody directed against annexin A1, and further incubated with beta cells (Fig. 4) . MP capture probed by the red fluorescence of beta cells was reduced by annexin A5 or by anti‐annexin A1 antibodies, whereas the FPR2/ALX antagonist had no effect (annexin A5: 22.5 ± 1 %; anti‐ANXA1: 29 ± 2 %, WRW4: 38 ± 1 % of PKH26^+^cells *versus* 40.5 ± 2 in control untreated cells, *n* = 4). These data are strongly suggestive of phosphatidylserine‐ and ANXA1‐dependent mechanisms governing the eMP integration in the target beta cells.

**Figure 5:**
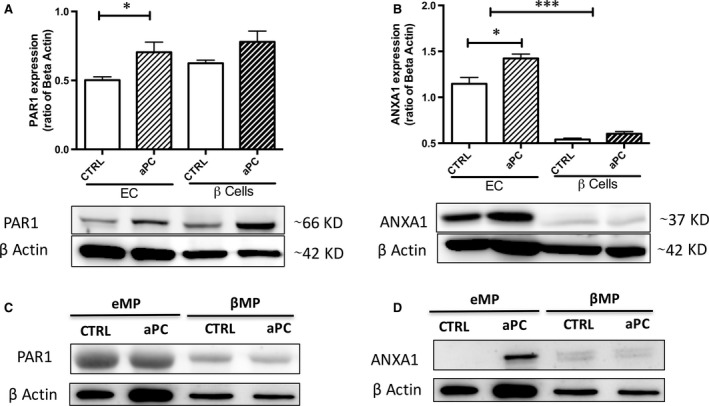
Expression of annexin A1 and PAR‐1 in eMP_aPC._ After 70 nM aPC treatment during 24 hrs, endothelial cells and β cell‐derived MP were harvested from supernatants, washed and concentrated. Expression of PAR‐1 (**A, C**) and annexin A1 (**B, D**) were assessed by Western blot in cell (**A, B**) (*n* = 4) and MP lysates (**C, D**) (*n* = 3). Data expressed as mean ± S.E.M. (aPC, activated protein C (Xigris^®^); CTRL, untreated cells; βMP, β‐cell derived MP; EC, endothelial cells; eMP, endothelial MP; MP, microparticles; PhSer eq., phosphatidylserine equivalent. *P < 0.05 *versus* CTRL ***P < 0.001 *versus* CTRL).

Interestingly, the basal expression of ANXA1 was barely detectable by Western blot in beta cells but was significantly higher in ECs. aPC up‐regulated the endothelial ANXA1 by about 20% (Fig. 5B, *P* < 0.05). The eMP_aPC_ ANXA1 content was significantly higher than that of eMP_CTRL_ (Fig. 5D, *n* = 3). At the opposite, the basal PAR‐1 expression in beta and ECs was comparable and was slightly enhanced by aPC. The PAR‐1 content of eMP_aPC_ and eMP_CTRL_ was similar.

### eMP protect beta cells by EPCR/PAR‐1 and ANXA1/FPR2 pathways

**Figure 6:**
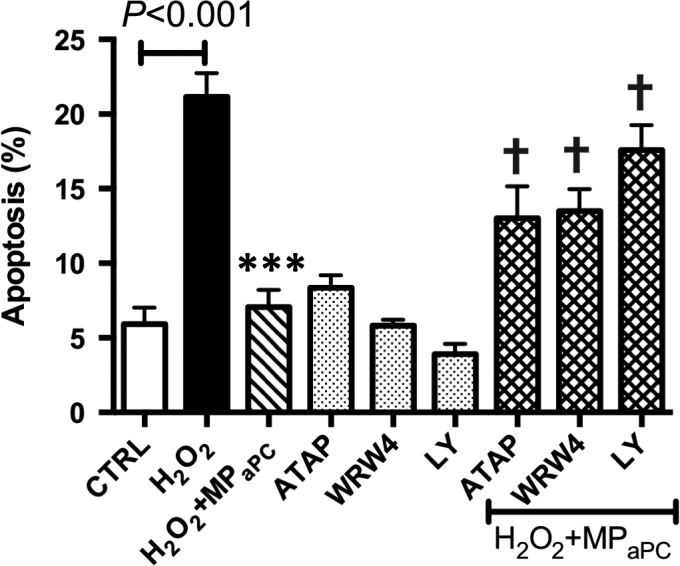
The protective effects of eMP_aPC_ are mediated through the PAR‐1/EPCR and ANXA1/FPR2 pathways. Apoptosis of β‐cells submitted or not to oxidative stress and MP was assessed by flow cytometry. β‐cells were pre‐incubated with a PI3K inhibitor (LY 294002, 10 μM, 1 hr) or FPR2 antagonist (WRW4, 10 μM, 1 hr) or a PAR‐1 antibody (ATAP2, 10 μg/ml) before a 6‐hrs treatment by MP and H_2_O_2_ stimulation. Data expressed as mean ± S.E.M., *n* = 4. (CTRL, untreated cells; MP_aPC_, endothelial microparticles released by aPC treatment. ***P < 0.001 *versus* H_2_O_2_; †*P* < 0.001 *versus* H_2_O_2_+ MP_aPC_).

To further investigate the pathways involved in eMP‐mediated beta cell cytoprotection, the cells were treated with different modulators (Fig. 6). Pre‐incubation with a PAR‐1 neutralizing antibody (ATAP2, SantaCruz, USA) partially reversed the eMP_aPC_‐driven protective effect, and led to enhanced apoptosis (eMP_aPC :_7.1 ± 1% *versus* 13 ± 2.1% in eMP_aPC_+ATAP2) suggesting a PAR1‐dependent pathway. Similarly, pre‐incubation of β‐cells with WRW4 (10 μM) partially reversed the eMP_aPC_ cytoprotective effect. In addition, LY294002, a PI3 kinase inhibitor, totally abolished the eMP_aPC_‐driven cytoprotection with a degree of apoptosis similar to the values observed in H_2_O_2_‐challenged cells. These data suggest that PI3 kinase is a common step to the PAR‐1 and annexin A1‐mediated effects (eMP_aPC_ : 7.1 ± 1%; eMP_aPC_+ LY294002: 17.6 ± 1.7%; H_2_O_2_‐treated cells: 21.2 ± 0.7%).

### eMP_aPC_ up‐regulate FPR2/ALX expression in β‐cells

**Figure 7:**
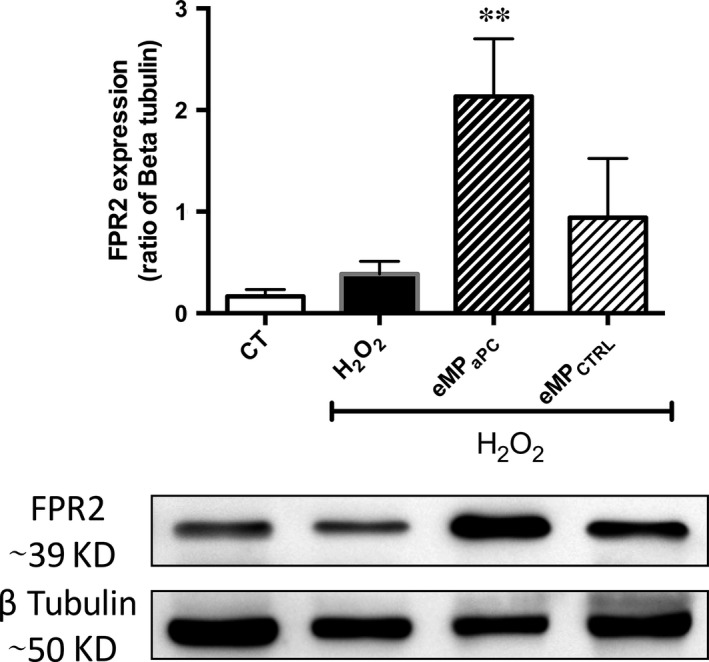
Expression of FPR2/ALX in eMP_aPC_ ‐ and eMP_CTRL_‐treated β‐cells submitted to H_2_O_2_. β‐cells were pre‐incubated for 12 hrs with 20 nM eMP_aPC_ or eMP harvested from untreated cells before addition of H_2_O_2_. FPR2/ALX expression was evidenced by Western blot in cell lysates after 24 hrs. Data expressed as mean ± S.E.M. (CTRL, untreated cells; FPR2, formyl peptide receptor 2; eMP_aPC_, endothelial microparticles released by aPC treatment; eMP_CTRL_, endothelial microparticles harvested from unstimulated cells. **P < 0.01 *versus* H_2_O_2_).

To confirm the contribution of FPR2/ALX, we analysed its expression in H_2_O_2_‐treated β‐cells after 6 hrs. Whereas FPR2/ALX expression was low in control untreated and H_2_O_2_‐treated β‐cells, Western blots showed that 20 nM eMP_aPC_ prompted a significant sevenfold up‐regulation of FPR2/ALX, suggesting a MP‐driven effect (*P* < 0.01, *n* = 4, Fig. 7).

### eMP_aPC_ reduce markers of oxidative stress and apoptosis in β‐cells

**Figure 8:**
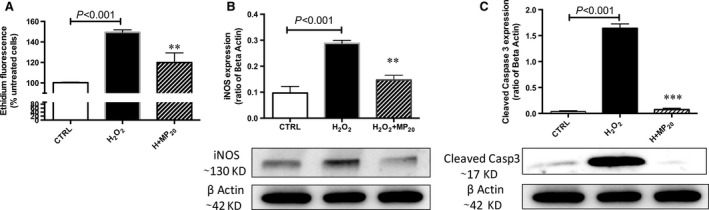
Effects of eMP_aPC_ on H_2_O_2_‐induced β‐cells apoptosis and oxidative stress. (**A**) β‐cells were pre‐incubated with 20 nM eMP_aPC_ during 12 hrs before addition of H_2_O_2_. Cells were incubated with the red probe dihydroxylethidium during 30 min. and red fluorescence was analysed by flow cytometry (*n* = 4). (**B, C**) β‐cells were pre‐incubated with 20 nM eMP_aPC_ during 12 hrs before addition of H_2_O_2_ (*n* = 4). iNOS (**B**) and cleaved caspase 3 (**C**) were evidenced by Western blot in cell lysates harvested after 24 hrs. Data expressed as mean ± S.E.M. (CTRL, untreated cells; iNOS, inducible nitric oxide synthase; MP_aPC,_ endothelial microparticles released after aPC treatment. **P < 0.01 *versus* H_2_O_2_; ***P < 0.001 *versus* H_2_O_2_).

We examined the oxygen species (ROS) content in eMP_aPC_‐treated cells, in the presence of H_2_O_2._ The amount of ROS by DHE fluorescence was decreased by 20 nM eMP_aPC_ (149.4 ± 2.5 in H_2_O_2‐_treated cells *versus* 120 ± 4.7 in H_2_O_2_+ MP treated cells (A.U. *P* < 0.01, *n* = 4, Fig. 8A). Similarly, the expression of inducible NO synthase (iNOS) assessed by Western blot returned to baseline, suggesting its prime role in the eMP_aPC_‐driven counteracted ROS generation. In addition, pro caspase‐3 activation prompted by H_2_O_2._ was limited in the presence of eMP_aPC_, as shown by its reduced cleavage into active caspase 3 (*n* = 4, *P* < 0.001, Fig. 8C).

### eMP_aPC_ protect islets against oxidative stress‐induced apoptosis and restore islet function

**Figure 9:**
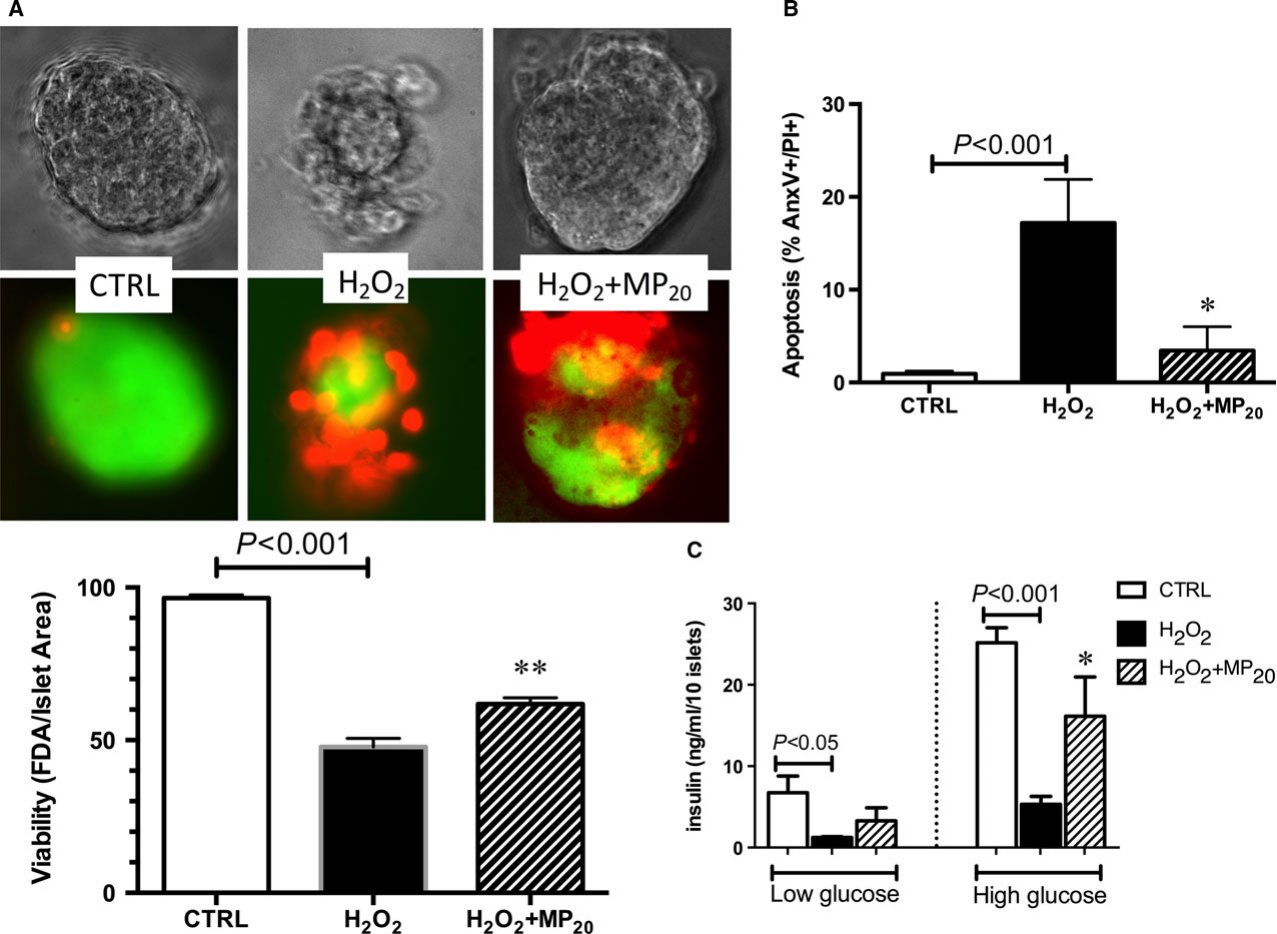
Effects of MP on islet viability, apoptosis and function. (**A**) Viability of islets. Rat islets were pre‐incubated with MP_aPC_ during 12 hrs before treatment by H_2_O_2_ during 6 additional hours a : Representative photographies of one islet per each condition. Following incubation, islets were stained by green FDA probe and red propidium iodide fluorescent probes. Red and green fluorescences were analysed after dissociation of the islet constitutive cells by flow cytometry following trypsin treatment. (**B**) Apoptosis : Cells of islets were stained by propidium iodide and annexin A5. Data expressed as mean ± S.E.M. (**C**) islets function assessed by insulin secretion after glucose challenge: Islets were pre‐incubated for 2 hrs in Krebs solution containing 2.5 mM glucose before incubation in Krebs buffer containing 25 mM glucose (high glucose). (CTRL, untreated cells; FDA, fluorescein diacetate; MP_aPC,_ endothelial microparticles released by aPC treatment. *P < 0.05 *versus* H_2_O_2_; **P < 0.01 *versus* H_2_O_2_).

The β‐cell cytoprotection by eMP_aPC_ observed above in Rinm‐5f was further confirmed in isolated islets incubated with 100 μM H_2_O_2_ during 4 hrs. eMP_aPC_ pre‐treatment limited the H_2_O_2_‐induced apoptosis measured by PI/AnV double staining of the dissociated constitutive islet cells using flow cytometry (H_2_O_2_: 17.2 ± 4.7 % *versus* H_2_O_2_+MP: 3.8 ± 2.8 %, unstimulated islets: 1 ± 0.3%, *P* = 0.03 *versus* H_2_O_2_, *n* = 4). Accordingly, increased viability using PI/FDA double labelling was also measured (H_2_O_2:_ 47.8 ± 2.8%, H_2_O_2_+MP: 61.8 ± 2%, unstimulated islets: 97.5 ± 1 %, *P* = 0.007 *versus* H_2_O_2_, *n* = 4). Importantly, when H_2_O_2_‐treated islets were challenged by high glucose concentration (25 mM), their ability to secrete insulin was restored by eMP_aPC_ (H_2_O_2:_ 5.3 ± 1; H_2_O_2_+MP: 16.1 ± 4.8; unstimulated: 25.2 ± 1.9, ng/ml insulin /10 islets, *P* = 0.04, *n* = 4, Fig. 9).

## Discussion

Our work demonstrates the protective effects of aPC‐generated endothelial MP on β‐cell function. The key finding of our data is that eMP_aPC_ convey EPCR together with aPC and act as paracrine β‐cell cytoprotective effectors, in line with previous observations of their autocrine cytoprotective effects 27. In the present study, we show that eMP_aPC_ deliver EPCR to target β‐cells and prompt an EPCR‐dependent PAR‐1 pathway within β‐cells. Interestingly, we evidenced that eMP_aPC_ cargo annexin‐A1 and active aPC and that MP interactions with the target cell are mediated through phosphatidylserine and ANXA1‐dependent mechanisms^22^. In addition, EPCR and FPR2/ALX expressions in target cells were up‐regulated by eMP_aPC_. The preservation of β‐cell survival and function by eMP_aPC_ was confirmed in isolated islets submitted to H_2_O_2_ to mimic the oxidative stress associated with ischaemia. Altogether, the study demonstrates that endothelial MP act as true mediators in cell crosstalk within the islets and confirms the key contribution of the endothelium for islet engraftment, survival and function of prime importance in islet‐transplanted patients 35.

### aPC favours the generation of endothelial MP able to prevent β‐cell dysfunction and apoptosis

Several studies have suspected a possible MP‐mediated effect on β**‐**cell function and survival. *Figliolini et al*. studying a suspension of MP and exosomes, also termed extracellular vesicles, proposed that they would contribute to the crosstalk between endothelial and β**‐**cells within the islets through the delivery of specific miRNA involved in β**‐**cell function and endothelial angiogenesis 17. We previously reported that MP shed from β‐cells in response to oxidative or cytokine stress induce the apoptosis of naive β**‐**cells 13 and that eMP isolated from aPC‐treated septic rats restored the vascular tone and protect cardiac and vascular tissues from NF‐κB and cyclooxygenase‐2 activation 26. Extracellular vesicles derived from endothelial progenitor cells were also reported protective in an islet model 20. They also reduced ischaemia–reperfusion injury in rats by transferring pro‐angiogenic miRNA 36, 37.

In the present study, we accordingly evidenced limited apoptosis in β**‐**cells treated by endothelial‐derived MP that was highly enhanced when MP were harvested from aPC‐treated cells (Fig. 1). These observations of the eMP_aPC_‐mediated effects were confirmed in eMP_aPC_‐treated islets submitted to H_2_O_2_ (Fig. 9) and were independent from the presence of exosomes (Fig. 1D).

### eMP_aPC_ deliver EPCR and mediate PAR‐1‐dependent cytoprotective signalling

Our data extend the previous observation that eMP_aPC_ transfer of EPCR and convey aPC to target ECs 27 (Fig. 2). We showed that eMP_aPC_ also target β‐cells cytoprotection and relies at least in part on PAR‐1‐ and PI3‐kinase‐dependent pathways. Specific inhibition of PI3 kinase totally abolished cytoprotection (Fig. 6), in line with the reported pI3K/Akt‐dependent cytoprotective pathway initiated by the aPC‐driven cleavage of raft‐localized PAR‐1 38. In our model, the transfer of EPCR/aPC may account for the MP‐mediated beneficial effects (Figs 8 and 9). However, the eventual role of a truly soluble form of the EPCR/aPC complex eventually cleaved from eMP_aPC_ by proteases present at the vicinity of the transplanted islet remains to be investigated *in vivo*. Indeed, in response to inflammatory and oxidative stress, endothelial EPCR is cleaved by TACE and shed as a soluble form in the perivascular environment, thereby blunting the cellular action of circulating aPC 39, 40. Under conditions of oxidative stress, our data indicate that eMP_aPC_ are beneficial actors still able to rescue damaged ECs through the delivery of the functional EPCR, aPC, PAR‐1 complex and of ANXA1, of eventual interest in the preservation of the intra‐islet endothelium (Fig. 1).

### Endothelial and β‐cell‐derived MP have distinct properties in response to aPC treatment

In our study, we could not detect any β**‐**cell cytoprotection against oxidative stress by aPC alone, although low EPCR expression was detectable as reported previously 41. In addition, βMP_aPC_ did not either protect β‐cells and 50 nM aPC even slightly increased apoptosis (Fig. 1).

The fact that Contreras *et al*. demonstrated aPC efficacy in the preservation of islets and liver endothelium after islet transplantation questions the underlying mechanisms 28. In our study, despite the fact that aPC prompted PAR‐1 expression in both β‐cells and ECs, EPCR concentration and ANXA1 remained poorly expressed in β‐cells and derived‐MP compared to their endothelial counterparts (Figs 3 and 5).

Conversely, we evidenced, enhanced aPC‐driven annexin A1 expression in ECs. In line with our data, the expression of various cytoprotective proteins as a Rac1, a PI3K‐dependent EPCR downstream event has been reported 42. Furthermore, an EPCR/PAR‐1‐dependent down‐regulation of sPLA2 was described by Bae *et al*. 43. One explanation could be brought by our observation of the EPCR‐driven up‐regulation of ANXA1, a well‐known PLA2 inhibitor 43. Therefore, ANXA1‐enriched eMP_aPC_ could account at least in part for the anti‐inflammatory and anti‐oxidative responses in target β**‐**cells and islets.

Other observations of the preservative action of aPC on β‐cells through the fine‐tuning of immunosuppressive Treg in type 1 diabetes were associated with the up‐regulation of EPCR on beta cells 41. Our data showing a direct eMP_aPC_‐driven modulation of the pro‐inflammatory and pro‐apoptotic responses of β‐cells are in line with these.

### eMP_aPC_ contain annexin A1 and mediate FPR2/ALX‐dependent pathways in β‐cells

As previously described in ECs, we found that PAR‐1 inhibition only leads to the partial loss of the eMP_aPC_ cytoprotective effect in β**‐**cells 27. In addition, Perez‐Casal showed that aPC harboured by EPCR^+^‐eMP was more efficient than aPC alone in cardiovascular cytoprotection and angiogenesis 42, an observation that was confirmed by our finding that eMP_aPC_ expose higher aPC activity and greater amounts of EPCR (Figs 2 and 3) 21, 22, 44, 45, 46

Our data bring a bunch of evidences strengthening the hypothesis of an alternate eMP_aPC_‐mediated ANXA1‐dependent pathway in β‐cells (Figs 4, 5, 6, 7). Indeed, eMP_aPC_ reduced apoptosis, enhanced viability and restored insulin secretion under conditions of oxidative stress (Fig. 9), in line with the reported observation that ANXA1 directly enhances islet β‐cell secretory function 47, 48. Our data indeed confirm previous reports describing MP‐embedded annexin A1 as a contributor to the MP capture by target cells and to downstream events 22. Indeed, ANXA1‐ containing endothelial MP were proven beneficial through the transfer of either proteins, RNAs or miRNA to target cells 22, 35.

The previous observation that ANXA1 effects persisted after its removal from the culture medium 46 is consistent with our data showing that eMP_aPC_ exert β‐cell cytoprotection through the up‐regulation of FPR2/ALX and PI3‐kinase, a downstream event previously described in the inflamed endothelium (Fig. 6) 45. In our hands, the ANXA1 content of endothelial MP was negligible when they were harvested from unstimulated ECs, whereas it was greatly enhanced by aPC treatment (Fig. 5).

Because FPR2 inhibition by the WRW4 antagonist only partially limited the eMP_aPC_‐driven protection, it is tempting to speculate that ANXA1 exerts indirect effects through multiple mechanisms, among which ANXA1 binding to the phospholipids of the β‐cell plasma membrane, as already reported in the presence of exogenous soluble ANXA1 23, 24, 25. However, our data demonstrate the up‐regulation of FPR2/ALX by eMP_aPC_ in target β‐cells that would also have amplify the cell response to eMP_aPC_ 49.

Our demonstration of an eMP‐driven cytoprotection that is FPR2/ALX and phosphatidylserine dependent is consistent with a pioneered work showing that ANXA1 binds to pancreatic islets in a calcium‐dependent and independent manner, highly suggestive of both receptor‐ and phospholipid‐mediated mechanisms 47.

Altogether, in our hands, ANXA1 appeared pivotal in the eMP_aPC_‐driven β**‐**cell cytoprotection through multiple pathways, namely enhancement of eMP capture, FPR2/ALX activation and its up‐regulation 21, 22.

Nevertheless, our data remain to be confirmed in animal models although several clues of the importance of ANXA1 in cytoprotection can be found in the literature. Indeed, ANXA1 is localized into insulin secretory granules of the islets and was suggested to modulate insulin secretion in an autocrine or paracrine manner 22, 46, 50. Under physiopathological conditions, the initial exposure of phosphatidylserine by apoptotic cells and MP shedding could be viewed as a protective response enabling the binding of the secreted ANXA1 51 of benefit through an anti‐inflammatory MP‐mediated crosstalk, eventually prompted by miRNA transfer 22, 35. Such a paracrine ANXA1 action was demonstrated for neutrophil‐derived MP that exert a powerful anti‐inflammatory effect on ECs *via* ANXA1 delivery 21.

**Figure 10:**
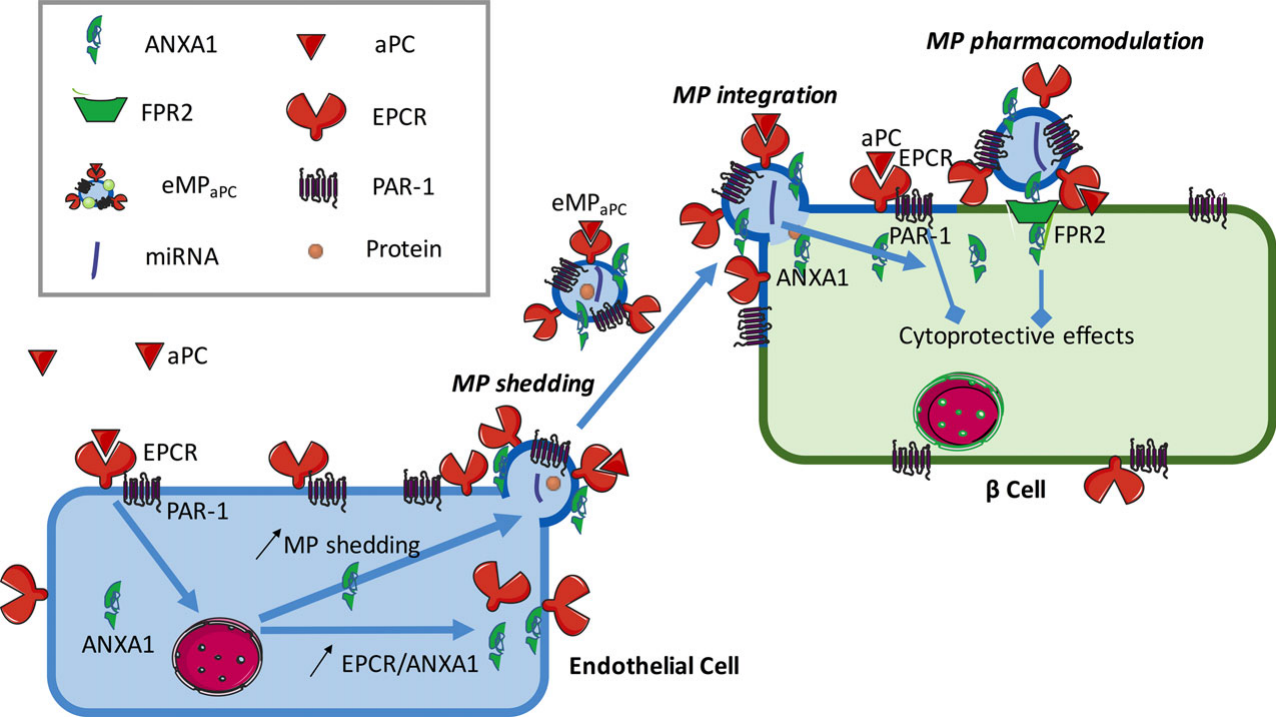
Hypothetical cytoprotective mechanisms triggered by endothelial microparticles released from aPC‐treated cells. The binding of aPC to its receptor, EPCR, causes the specific cleavage of PAR‐1 and activation of its dependent cytoprotective pathway in endothelial cell, thereby causing the up‐regulation of annexin A1 and the release of EPCR and annexin A1 enriched‐MP. These endothelial microparticles act by autocrine and paracrine pathways *via* the activation of the annexin A1 receptor, FPR2/ALX. They bind to target β‐cells in a phosphatidylserine and FPR2‐dependent manner and exert cytoprotection through the transfer of EPCR / PAR‐1 and downstream *via* the activation of FPR2/ALX and PAR‐1‐mediated pathways.

In summary, this study characterizes a new cytoprotective action exerted by endothelial MP on insulin‐secreting β‐cells that could be significantly enhanced by aPC with great potency in isolated islets submitted to major oxidative stress (Fig. 10). In our model, aPC protected both β‐cells and pancreatic islets through the release of endothelial aPC/EPCR^+^‐MP that behaved as cellular effectors in the preservation of islet function. Two different and non‐exclusive pathways initiated by such MP have been identified: one dependent on EPCR/PAR‐1 and the other pertaining to the delivery of ANXA1 and FPR2/ALX‐mediated responses. In the context of transplantation, MP may prove a promising therapeutic tool in the pre‐ and post‐conditioning of islet grafts, especially in view of the new engineered protein C that retain cytoprotective effects with minimal anticoagulant properties 4. Our data strongly support the ability of endothelial MP to convey endogenous or cytoprotective proteins that preserve islet function, limit the graft cell loss and favour islet re‐vascularization and engraftment.

## Author Contributions

GK wrote the manuscript and performed the main part of the experimental study. MK contributed to the design of improved islet isolation procedures and performed part of the islet experiments and realized the image analysis. AEH performed part of the ECs experiments and Western blot and PB contributed to the production of MP. MA and LA helped in the extraction of primary endothelial cell and performed Western blot and DHE measurements. BY measured the MP, and FZ and LA gave technical support for flow cytometry. FT, FZ and JBH gave support for the design of aPC activity measurement in MP and cells. JP contributed to flow cytometry analysis. FT, GUS and LK designed the study and FT, GUS and LK corrected the manuscript.

## Disclosure

The authors of this manuscript have no conflict of interest to disclose.
